# Aestivation induces widespread transcriptional changes in the African lungfish

**DOI:** 10.3389/fgene.2023.1096929

**Published:** 2023-01-17

**Authors:** Yuhan Niu, Lihong Guan, Cheng Wang, Haifeng Jiang, Guogang Li, Liandong Yang

**Affiliations:** ^1^ College of Life Sciences, Qinghai Normal University, Xining, Qinghai, China; ^2^ State Key Laboratory of Freshwater Ecology and Biotechnology, Institute of Hydrobiology, Chinese Academy of Sciences, Wuhan, China; ^3^ Academy of Plateau Science and Sustainability, Qinghai Normal University, Xining, China; ^4^ College of Life Sciences and Technology, Xinxiang Medical University, Xinxiang, Henan, China; ^5^ Key Laboratory of Biodiversity Formation Mechanism and Comprehensive Utilization of the Qinghai-Tibet Plateau in Qinghai Province, Qinghai Normal University, Xining, China

**Keywords:** lungfish, *Protopterus annectens*, aestivation, transcriptome, differential gene expression

## Abstract

Aestivation is a special ability possessed by some animals to cope with hot and dry environments utilizing dormancy. At a macroscopic level, dormant animals stop moving and eating. At the microscopic level, the expression of a large number of genes in these animals is strictly controlled. However, little is known about what changes occur during aestivation, especially in fish. In this study, we used transcriptome analysis to examine what changes occur in the gills and lungs of the African lungfish (*Protopterus annectens*) during the maintenance phase of aestivation and speculated on their causes. We found that aestivating transcriptomes were highly similar between gills and lungs. We also found that some genes showed differential expression or alternative splicing, which may be associated with different organs. In addition, differential expression analysis revealed that the lungs maintained significantly higher bioactivity during aestivation, which suggests that the main respiratory organ in aestivating lungfish can transform. Our study provides a reference point for studying the relationship between aestivation and hibernation and further increases understanding of aestivation.

## Introduction

As a living fossil, lungfish make up a long-existing vertebrate group, with extant lungfish taxa including *Neoceratodus forsteri* (Australian lungfish), *Lepidosiren paradoxa* (South American lungfish), *Protopterus aethiopicus*, *P. amphibius*, *P. annectens*, and *P. dolloi* (African lungfish) (Mlewa et al., 2011). Lungfish are different from other fish in that they have both gills and lungs. Their lung function has many obvious features in common with tetrapods, whereas their gills are similar to the physiological functions of other bony fish (Glass, 2011; Amemiya et al., 2013; Dan L. et al., 2013). Australian lungfish have a usable gill system and a single lung, whereas both South American and African lungfish have complex lungs and degenerate gills (Burggren & Johansen, 1986; Glass, 2011). African lungfish are obligate air breathers and can digest in mud cocoons during seasonal dry spells (Greenwood, 1986; Ballantyne & Frick, 2011; Mlewa et al., 2011). Under natural conditions, aestivation in *P. annectens* lasts seven or 8 months, depending on the length of the dry season (Greenwood, 1986; Mlewa et al., 2011).

African lungfish have been successfully induced into aestivation in the laboratory (DeLaney et al., 1974). Although both aestivation and hibernation are ostensibly states of dormancy, in aestivation the body’s metabolism slows under high ambient temperatures (Glass, 2011). Aestivation has been described as a state of behavioral inactivity and metabolic inhibition at high ambient temperatures, especially during the dry season, in terrestrial animals (Chew et al., 2015). It is divided into an induction period, a maintenance period, and an arousal period according to the different physiological processes that are occurring. Both invertebrates and vertebrates can enter a state of aestivation. Examples include invertebrates such as sea cucumber (*Apostichopus japonicus*), land snail (*Otala lactea*), and African malaria mosquito (*Anopheles gambiae*) (Brooks & Storey, 1990; Ramnanan & Storey, 2006; Lehmann et al., 2010; Chen et al., 2013), and vertebrates such as fish (eel, Canterbury mudfish, and lungfish), amphibians (frog and toad), reptiles (turtle), and mammals (mouse and squirrel) (Loong et al., 2008a; Loong et al., 2008b; Loong et al., 2012; Hiong et al., 2013).

Most studies of *P. annectens* during different periods of aestivation have been conducted at the proteomic level. Aestivating *P. annectens* has an intact gill morphology but is almost completely disabled during the maintenance phase and resumes nitrogen metabolism after awakening (Chng et al., 2016). A study of Rhesus glycoproteins showed that the gills cease functioning at the beginning of aestivation and recover during the recovery period (Chng et al., 2017b). Subsequent studies focused on changes in urea metabolism in aestivating lungfish successfully proved that urea plays an important role in maintaining osmotic pressure and water retention in the bodies of lungfish during aestivation and clearly described changes in aquaporin 1, aquaporin 3, and urea transporters in each period of aestivation (Chng et al., 2016; 2017a). Y *et al.* cloned myostatin in dormant lungfish and elucidated how lungfish resist muscle atrophy during aestivation (Ong et al., 2017). Chng *et al.* showed how Rhesus glycoproteins change during aestivation and found for the first time that Rhag was grouped closer to fishes, whereas Rhbg and Rhcg were grouped closer to tetrapods (Chng et al., 2017b). Finally, Garofalo *et al.* found that gill and lung molecular components of the Nitric Oxide Synthase/Nitric Oxide (NOS/NO) system change in a tissue-specific manner in parallel with organ readjustment in the gills and lungs of *P. annectens* (Garofalo et al., 2015).

The current proteomic study of African lungfish in the aestivation state details how the relevant proteins change during the dormant phase. It also provides a good theoretical basis for further understanding of aestivation. In addition, we found that the gills and lungs not only perform respiratory functions but also other important physiological functions such as urea metabolism during aestivation. Therefore, we suggest that the gills and lungs of African lungfish play a key role in maintaining the aestivation state, but we still do not know how they perform their functions at the molecular level. Therefore, in this study, we subjected the gills and lungs of *P. annectens* to transcriptome analysis and used bioinformatics to identify differentially expressed genes in the two organs during the maintenance phase of aestivation.

## Materials and methods

### Ethics statement

All procedures were performed following relevant guidelines and regulations and approved by the ethics committee of the Institute of Hydrobiology, Chinese Academy of Sciences.

### Lungfish

This study was performed on the African lungfish *P. annectens* (Order *Lepidosireniformes*; Family Protopteridae). Specimens of *P. annectens* (Body length 50–100 cm, adult individuals) were collected from Central Africa and imported through a neighborhood fish farm in Guangzhou, China. Samples were maintained in glass aquaria filled with dechlorinated water, containing 2.3 mmol L^−1^ Na^+^, 0.54 mmol l^−1^ K^+^, 0.95 mmol L^−1^ Ca^2+^, 0.08 mmol l^−1^ Mg^2+^, 3.4 mmol L^−1^ Cl^−^ and 0.6 mmol L^−1^ HCO_3_^−^, at pH 7.0°C and 25°C in the laboratory, and water was changed daily. No attempt was made to separate the sexes. Fish were acclimated to laboratory conditions for at least 1 month. During the acclimation period, the fish were fed frozen fish once a day while artificially simulating the duration and intensity of daylight saving time light at the origin of the samples.

### Experimental conditions and tissue sampling

Food was withdrawn 96 h before experiments for both control and aestivating lungfish, which gave sufficient time for the gut to be emptied of all food and waste (Chng et al., 2017b). Lungfish (N = 3) reared in freshwater were used as controls and euthanized with an excess of neutralized 0.05% MS222 for tissue sampling. Also stored to −80°C for backup. Some lungfish were induced to aestivate at 27°C–29°C and 85%–90% humidity individually in plastic tanks (L29 cm x W19 cm x H17.5 cm) containing 15 ml dechlorinated tap water (made 0.3‰ with seawater). It took approximately 6 days for the lungfish to be encased in a brown dried mucus cocoon and these 6 days were counted as part ofthe aestivation period. The lungfish were allowed to aestivate for 6 months. In order to maintain a high humidity (>90%) within the tank, 1–2 ml of water was sprayed onto the side of the tank daily. After 6 months of aestivation, the mud cocoons were broken by hand and the lungfish were euthanized using 0.05% MS222 neutralizer (N = 3 for each group). Acquired gill and lung samples were stored at −80°C.

### RNA extraction

Frozen tissue was homogenized in 1 mL TRIzol reagent (Takara, China). RNA was extracted according to the manufacturer’s instructions and treated with RNase-free DNase I (New England Biolabs) for 30 min at 37°C to remove residual DNA. The quality of the RNA was determined on 1.2% EtBr-agarose gels, and the quantity (A260/A280) was measured with a Nanodrop 2000 spectrophotometer (Thermo Scientific) and a 2100 Bioanalyzer (Agilent Technologies).

### Preparation and sequencing of cDNA libraries

The NEBNext poly(A) mRNA Magnetic Isolation Module (NEB, E7490) was used to purify poly(A) mRNA from total RNA. Then cDNA libraries were prepared with the NEBNext mRNA Library Prep Master Mix Set for Illumina (NEB, E6110) and NEBNext Multiplex Oligos for Illumina (NEB, E7500). We used 1.8% EtBr-agarose gels to measure the lengths of fragments in the cDNA libraries. qPCR was performed using the Library Quantification Kit-Illumina GA Universal (Kapa, KK4824). Qualified cDNA libraries were then immobilized in an Illumina cBot to generate clusters and sequenced with an Illumina HiSeqTM 2500 (Illumina) at Biomarker Technologies Co., Ltd. The raw sequencing data were uploaded to NCBI (accession no. PRJNA903405). In addition, to ensure the accuracy of the subsequent assessment of differential expression between the gills and lungs of African lungfish, we performed stratified clustering and principal component analysis.

### Analysis of RNA-seq data

We used FastQC (version 0.11.9) to check the quality of the raw RNA-seq reads. Only paired-end reads that were longer than 50 bp at either end after trimming were ultimately retained for subsequent analysis. High-quality paired reads from each sample were compared to the African lungfish reference genome (NCBI Taxonomy ID: 7888) on NCBI (https://www.ncbi.nlm.nih.gov/data-hub/genome/GCF_019279795.1/) with hisat2 (version 2.2.1). Data were assembled with StringTie (version 2.2.1), and raw count values were calculated with its script prepDE.py. Ballgown (version 2.28.0) was used to estimate the abundance of transcripts (fragments per kilobase million) (Pertea et al., 2016). Differentially expressed transcripts (|Log2FoldChange| ≥ 1, *p*-value <0.05) in samples were screened with the R package DESeq2 (version 1.36.0) (Liu et al., 2021).

### Enrichment analysis

Data on differential expression were submitted to EggNog for online annotation (https://eggnog-mapper.embl.de/). Based on the results of the online annotation and the African lungfish reference genome, we built a database for African lungfish enrichment analysis using the R package AnnotationForge (version 1.38.1) and performed Gene Ontology (GO) and Kyoto Encyclopedia of Genes and Genomes (KEGG) enrichment analyses of gill and lung sample data, respectively (*p*-value <0.05).

### Characterization of alternative splicing events

ASEs are divided into five broad categories: skipped exon (SE), alternative 5′splice site (A5SS), alternative 3′splice site (A3SS), mutually exclusive exons (MXE), and retained intron (RI) (Barbazuk et al., 2008). We used rMATS (Shen et al., 2014) to detect and calculate the five types of ASEs. rMATS can identify these ASEs from GTF files with annotated transcripts and calculate the number of reads corresponding to each. We used *p* < 0.05 as a threshold for the detection of differential alternative splicing events in experimental and control gill and lung tissue.

### Quantitative real-time PCR

cDNA samples were generated from mRNAs of the lungs of the two lungfish groups with reverse transcriptase (Promega) in the presence of an RNase inhibitor (Invitrogen) with a cocktail of oligoT and dNTP (Takara, China). To validate the expression patterns discovered in our RNA-seq analyses, we performed qRT-PCR using SYBR Green (Roche) chemistry on a Light Cycle^®^ 480 II (Roche). We used Primer Premier 5 to design real-time PCR primers based on sequences derived from our assembled lungfish transcriptome (Van Beers et al., 1998). Three replicates for each gene were analyzed, with beta-actin serving as an internal control. The PCR cycling program consisted of 45 three-step cycles of 10 s/95°C, 10 s/TA, and 20 s/72°C. To confirm signal specificity, we ran a melting program after the PCR cycles were completed. Student’s t-test was used to detect differences in gene expression between the two different samples.

## Results

### Transcriptome data

A total of 12 cDNA libraries were constructed in this study. Based on mRNA sequencing, we obtained 8.25 GB of sequence in the gills of the control group and 9.18 GB of sequence in the lungs. Similarly, we obtained 6.25 GB of sequence in the gills of the experimental group and 6.45 GB of sequence in the lungs. Among these 12 data sets, 88.67%–93.13% of reads could be successfully mapped to annotated regions in the African lungfish reference genome, with an average mapping rate of 91.44%. This indicates the good quality of the data reads obtained (Supplementary Table S1).

### Analysis of differential expression

**FIGURE 1:**
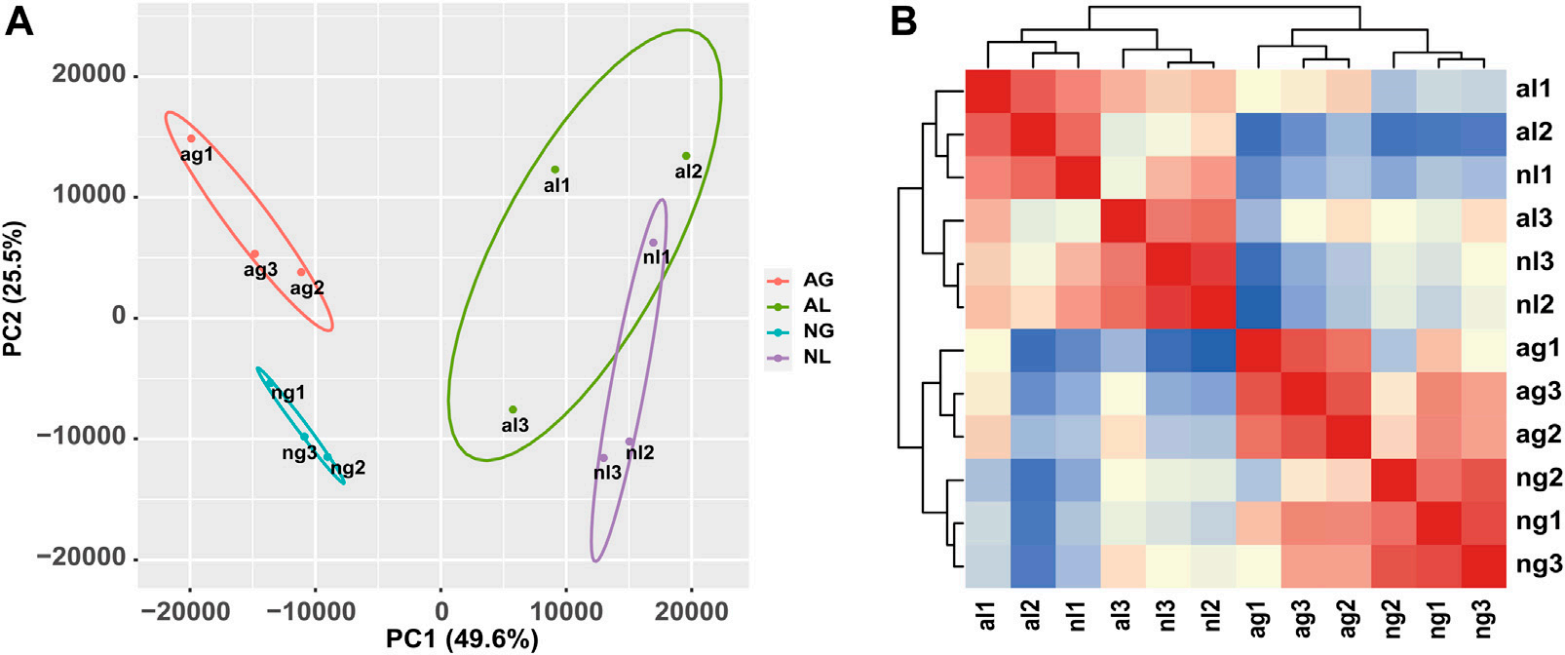
Analysis of African lungfish samples during aestivation. **(A)** Heat map of cross-correlations of all samples with differentially expressed transcripts. **(B)** Cluster diagram of principal component analysis for all samples. ag: aestivating gills; ng: non-aestivating gills; al: aestivating lungs; nl: no-aestivating lungs.

The results of hierarchical clustering between samples and principal component analysis (Figures 1A, B) showed that the samples exhibited relatively good clustering by tissue and state, which indicates good biological replication and credible data analysis. To gain insight into the differential expression between organs during aestivation, we detected a total of 2450 transcripts in gill tissue showing obvious upregulation and 3245 transcripts in gill tissue showing obvious downregulation after aestivation.

**FIGURE 2:**
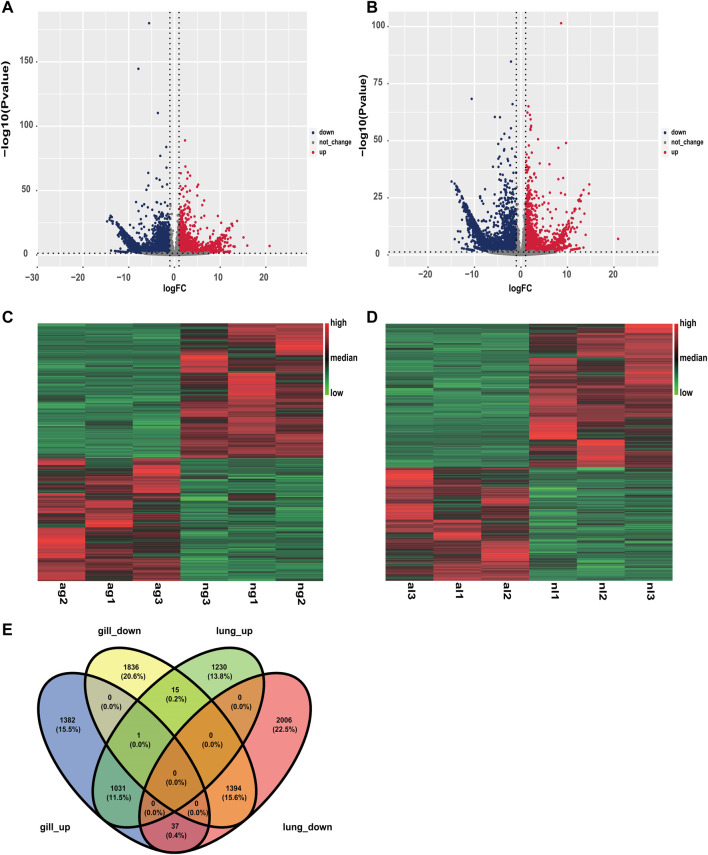
Differences in expression in the organs of aestivating African lungfish. **(A)** Volcano map of differentially expressed transcripts in the gills of aestivating lungfish. **(B)** Volcano map of differentially expressed transcripts in the lungs of aestivating lungfish. **(C)** Heat map of differentially expressed transcripts in the gills of aestivating lungfish. **(D)** Heat map of differentially expressed transcripts in the lungs of aestivating lungfish. **(E)** Venn map of differentially expressed transcripts in the gills and lungs of aestivating lungfish.

Similarly, obvious up- and downregulation were detected in 2276 and 3437 transcripts, respectively, in lung tissue (Supplementary Tables S2, S3; Figures 2A, B). However, when we used a false discovery rate threshold of 0.05, only 5635 and 5657 transcripts remained differentially expressed in gills and lungs (Figures 2C, D). We found that 1031 (11.5%) and 1394 (15.6%) transcripts were jointly up- and downregulated, respectively, in both organs during aestivation compared to non-aestivation (Figure 2E). The analysis showed that, in addition to genes related to metabolism and cell proliferation, genes related to immune and inflammatory responses were significantly differentially expressed (Tables 1; Tables 2). This included, for example, the genes *NPM1* (nucleophosmin 1) and *RFFL* (ring finger and FYVE-like domain containing E3 ubiquitin protein ligase).

**TABLE 1:** Select differentially expressed genes in gills.

Transcription	Symbol	Description	log2FC	p-Value	FDR
XM_044069097.1	NPM1	nucleophosmin 1	1.051	1.110 × 10^−22^	1.660 × 10^−20^
XM_044069094.1	NPM1	nucleophosmin 1	1.338	1.170 × 10^−8^	2.290 × 10^−7^
XM_044082276.1	LOC122810711	bromodomain-containing protein 4-like	2.309	2.450 × 10^−20^	2.680 × 10^−18^
XM_044082336.1	LOC122810775	transcription activator BRG1-like	1.123	2.040 × 10^−10^	5.430 × 10^−9^
XM_044074048.1	RFFL	The ring finger and FYVE-like domain containing E3 ubiquitin protein ligase	1.142	6.340 × 10^−3^	2.74 × 10^−2^
XM_044087274.1	LOC122815022	leucine-rich repeat serine/threonine-protein kinase 2-like	−3.079	2.900 × 10^−10^	7.48 × 10^−9^
XM_044087069.1	LOC122814494	integrin alpha-M-like	−2.653	1.010 × 10^−4^	8.110 × 10^−4^
XM_044081128.1	LOC122809547	macrophage migration inhibitory factor-like	−6.185	1.810 × 10^−4^	1.350 × 10^−3^

**TABLE 2:** Select differentially expressed genes in the lungs.

Transcription	Symbol	Description	log2FC	p-Value	FDR
XM_044069097.1	NPM1	nucleophosmin 1	1.000	2.360 × 10^−9^	5.390 × 10^−8^
XM_044069094.1	NPM1	nucleophosmin 1	1.056	7.800 × 10^−4^	4.910 × 10^−3^
XM_044082276.1	LOC122810711	bromodomain-containing protein 4-like	1.754	1.540 × 10^−26^	3.380 × 10^−24^
XM_044074048.1	RFFL	The ring finger and FYVE-like domain containing E3 ubiquitin protein ligase	1.259	4.519 × 10^−1^	6.577 × 10^−1^
XM_044087069.1	LOC122814494	integrin alpha-M-like	−2.150	2.200 × 10^−85^	3.150 × 10^−81^
XM_044087274.1	LOC122815022	leucine-rich repeat serine/threonine-protein kinase 2-like	−3.604	1.780 × 10^−29^	5.610 × 10^−27^
XM_044081128.1	LOC122809547	macrophage migration inhibitory factor-like	−5.177	1.560 × 10^−3^	8.830 × 10^−3^

### GO enrichment and KEGG enrichment

Among the 982 statistically significant GO terms identified (*p*-value <0.05), we found that upregulated genes were more abundant in RNA-related processes (e.g., GO:0006397, GO:0008380, GO:0000377, GO:0000398; Figure 3A), whereas downregulated genes were more abundant in material transport and developmental control (e.g., GO:0035220, GO:0048563, GO:0090130, GO:0048737; Figure 3B). The lungs had significantly more upregulated differentially expressed genes than the gills during aestivation (Supplementary Table S4). This suggests that the main respiratory organ of the African lungfish undergoes a dramatic transformation during aestivation. Meanwhile, upregulation of p53 binding genes was observed in gills (GO:0002039), whereas upregulation of cell cycle protein regulatory genes was observed in lungs (GO:1904029, GO:0000079). These genes play important roles in regulating cell division processes, which suggests that the gills and lungs of lungfish might have different ways of regulating cellular value-added processes during aestivation.

**FIGURE 3:**
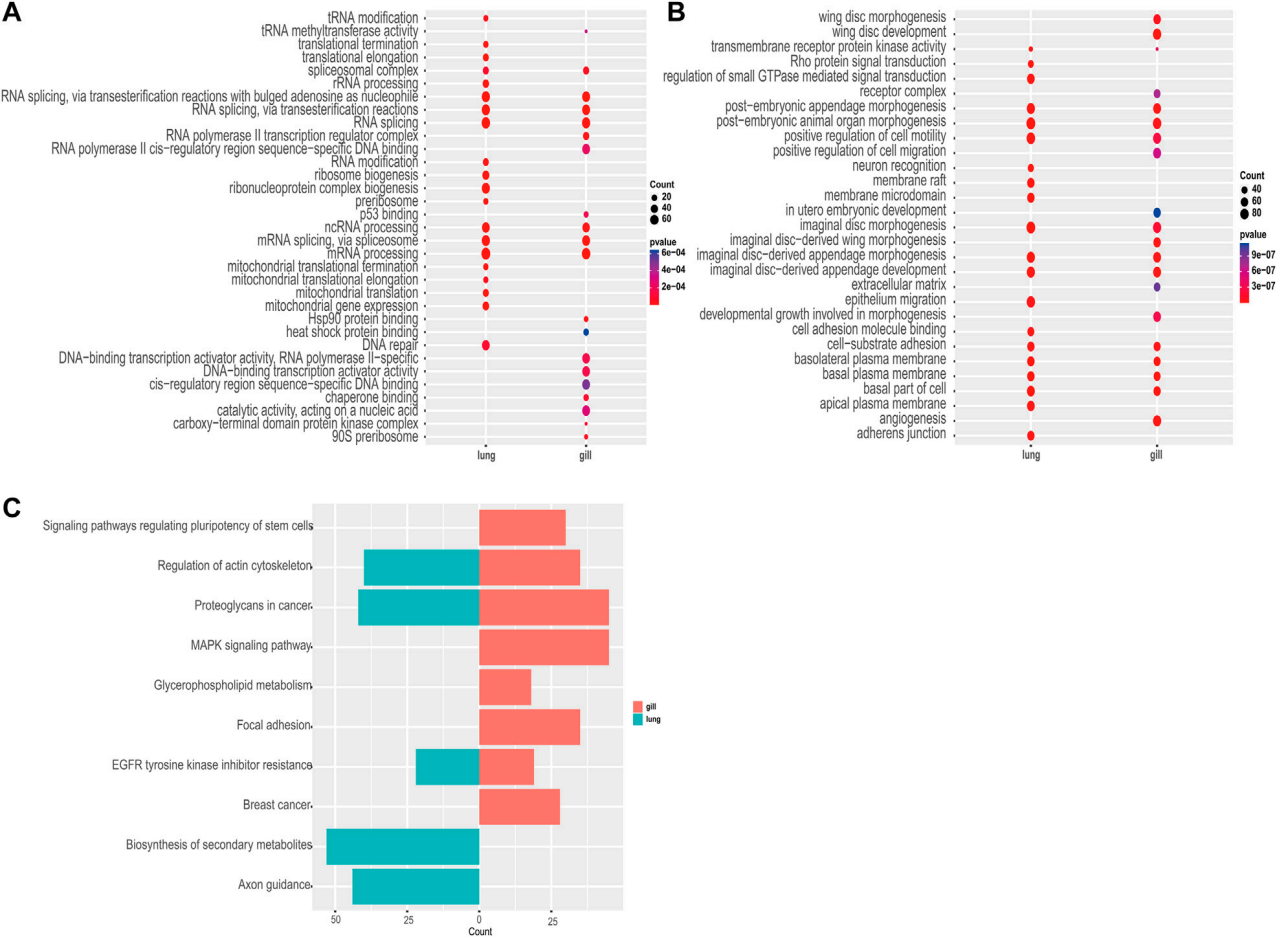
Enrichment analysis. **(A)** GO enrichment analysis of commonly upregulated genes in aestivating lungfish. **(B)** GO enrichment analysis of commonly downregulated genes in aestivating lungfish. **(C)** KEGG enrichment analysis of commonly downregulated genes in aestivating lungfish.

Next, we performed a KEGG enrichment analysis. Although we were not able to obtain good KO enrichment results in the upregulated pathway in our samples, the KO pathway enriched in the downregulated pathway was very indicative of the various physiological activities that occur in African lungfish during aestivation. We found that biosynthesis, cell differentiation, lipid metabolism, and other pathways (ko04550, ko00564, ko01110) were differentially inhibited in the enriched downregulated pathways in different organs. We also observed that in addition to significant inhibition of cell growth and cell motility (ko01521, ko04810), both gills and lungs exhibited significant downregulation of cancer-related glycoprotein pathways (ko05205), which may inhibit carcinogenesis. These enrichment results suggest that African lungfish reduce body energy expenditures during aestivation by suppressing physiological activities at all levels while indirectly increasing their capacity to suppress cancer.

### Alternative splicing events between gill and lung in aestivating African lungfish

To investigate the effect of aestivation on ASEs in the gills and lungs of African lungfish, we identified ASEs, including skipped exon SE, A5SS, A3SS, MXE, and RI, between aestivating and non-aestivating African lungfish.

**FIGURE 4:**
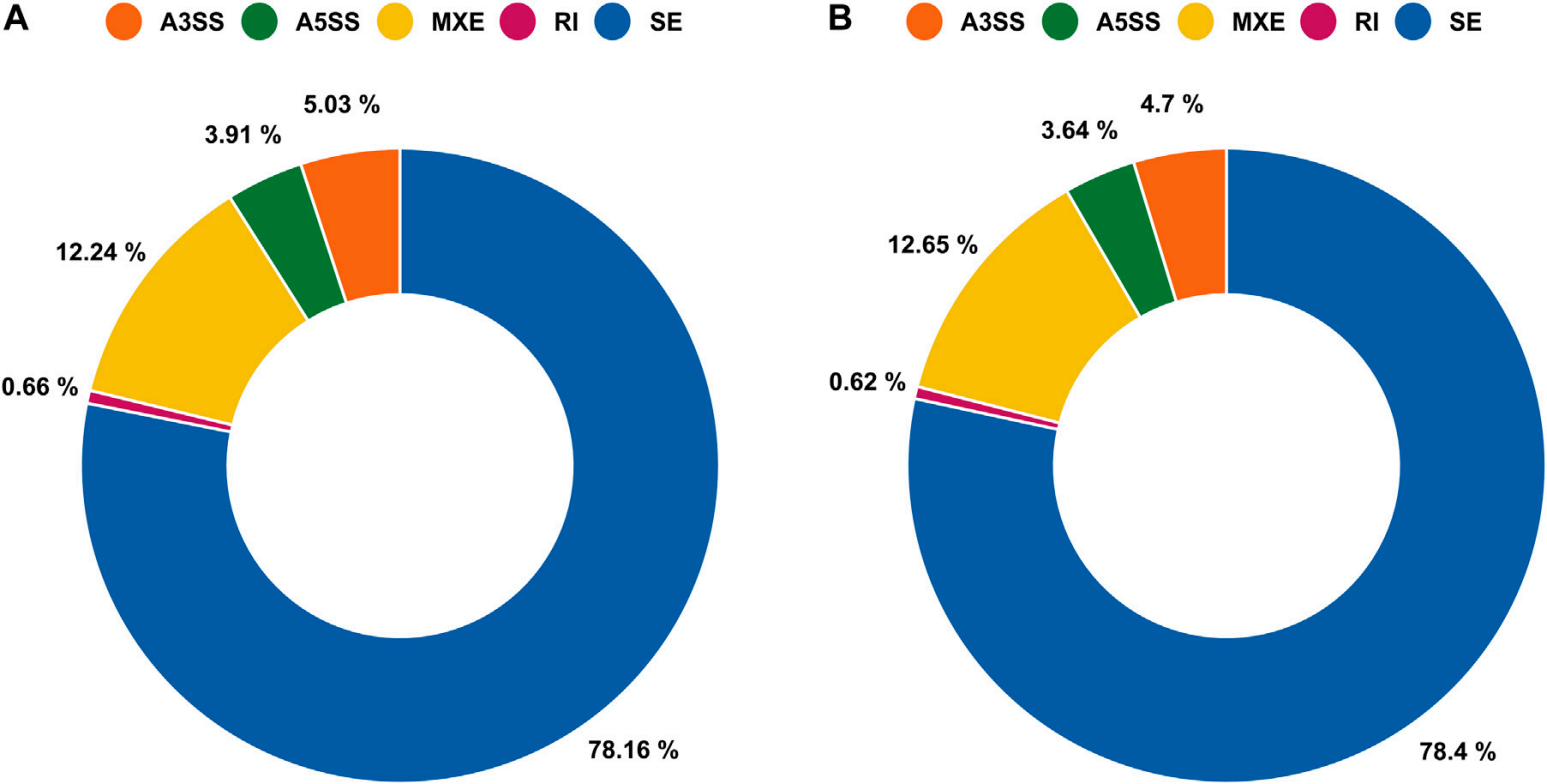
Alternative splicing events (ASEs) between gills and lungs of aestivating lungfish. **(A)** Circle chart showing the percent distribution of ASEs in gills. **(B)** Circle chart showing the percent distribution of ASEs in lungs.

A total of 29,905 ASEs were detected in the gills and 32,058 ASEs were detected in the lungs. The most abundant ASEs in both organs were SE (Supplementary Table S6). SE accounted for 78.16% of all ASEs in gills, followed by MXE (12.24%), A3SS (5.03%), A5SS (3.91%), and RI (0.66%; Figure 4A). SE accounted for 78.40% of all ASEs in lungs, followed by MXE (12.65%), A3SS (4.70%), A5SS (3.64%), and RI (0.62%; Figure 4B). *p* < 0.05 was used to confirm ASEs that differed between the two organs of African lungfish during aestivation (Supplementary Table S5). A total of 4528 significantly different ASEs were identified in gill tissue during aestivation: 2572 SE, 231 A5SS, 271 A3SS, 1429 MXE, and 25 RI; 3739 significantly different ASEs were identified in lung tissue during aestivation: 1879 SE, 165 A5SS, 176 A3SS, 1504 MXE, and 15 RI (Table 3). Taken together, these results suggest that the large number of ASEs in lungfish can effectively respond to some of the survival stresses experienced during aestivation.

**TABLE 3:** Select differential alternative splicing events.

Organ	Type	Gene Symbol	Description	p-Value	FDR
Gill	A3SS	TP73	tumor protein p73	8.840 × 10^−12^	1.420 × 10^−3^
Gill	A5SS	MYCL	MYCL proto-oncogene, bHLH transcription factor	4.490 × 10^−3^	2.434 × 10^−2^
Gill	MXE	CDIP1	cell death-inducing p53 target 1	1.930 × 10^−10^	2.260 × 10^−9^
Gill	SE	TPT1	tumor protein, translationally-controlled 1	3.340 × 10^−12^	3.600 × 10^−10^
Gill	RI	ABL2	ABL proto-oncogene 2, non-receptor tyrosine kinase	2.210 × 10^−3^	1.380 × 10^−2^
Lung	A3SS	LOC122790735	TAR DNA-binding protein 43-like	8.640 × 10^−7^	2.200 × 10^−5^
Lung	A5SS	SH3KBP1	SH3 domain-containing kinase binding protein 1	2.080 × 10^−3^	1.335 × 10^−2^
Lung	MXE	CDIP1	cell death-inducing p53 target 1	5.650 × 10^−13^	8.800 × 10^−12^
Lung	SE	TSG101	tumor susceptibility 101	3.900 × 10^−4^	6.310 × 10^−3^
Lung	RI	USP40	Ubiquitin-specific peptidase 40	2.170 × 10^−3^	1.776 × 10^−2^

### Quantitative RT-PCR analysis

**FIGURE 5:**
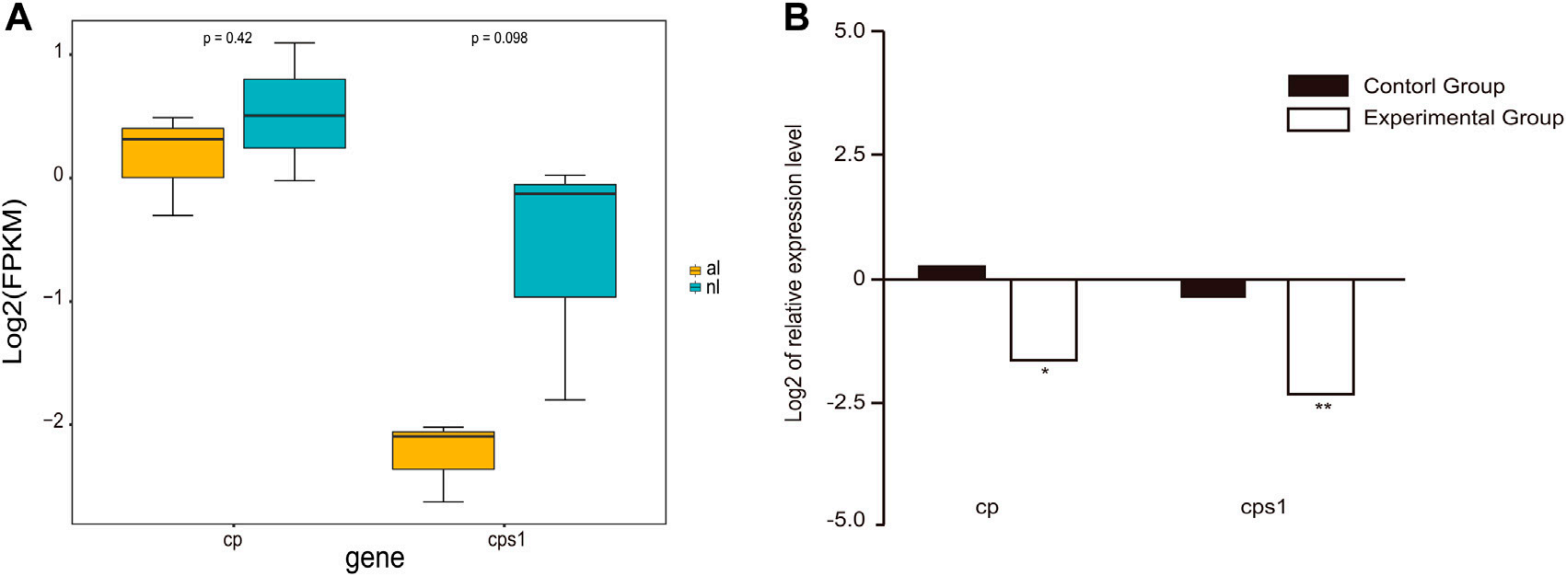
Gene expression in the lungs. **(A)** Expression of cp and cps1 genes in the lungs of non-aestivating and aestivating lungfish. **(B)** Validation of RNA-seq data using quantitative real-time PCR (qRT-PCR).

Gene expression analyses demonstrated a dramatic difference in expression between the lungs of non-aestivating and aestivating *P. annectens*. To validate the expression results of RNA-seq, we used qRT-PCR to quantify the expression of ceruloplasmin (*cp*) and carbamoyl-phosphate synthase 1 (*cps1*). The results showed that the expression of these genes was highly consistent between RNA-seq and qRT-PCR (Figure 5). Both were downregulated, which indicates the reliability of our RNA-seq data set.

## Discussion

Aestivation has received little attention in the past decades, but as research on hibernation continues, this state of dormancy, which is phenotypically similar to hibernation, is gaining attention. Both aestivation and hibernation are aerobic hypometabolic states, but aestivation is the evolutionary result of some organisms tolerating unfavorable conditions in arid environments (Storey, 2022). In addition, aestivation is associated with food deficiencies and involves the downregulation of metabolism (Glass, 2011). In this study, we used RNA-seq to obtain the first transcriptional profiles of the gills and lungs of aestivating and non-aestivating African lungfish. And in this way, we tried to explore the molecular changes that occur in African lungfish during the maintenance phase of aestivation. By comparing the gene expression of three different biological replicate samples from different states of each organ, we obtained highly correlated samples, which indicates that the subsequent differential expression analysis was reliable.

### Characteristics of organ changes during aestivation

African lungfish adapt to the rapidly changing environmental conditions experienced during their life cycle by making well-coordinated morphofunctional adjustments in multiple organs (Icardo et al., 2008). In our experiments we found that the lungs of aestivating lungfish undergo physiological hypertrophy accompanied by an increase in blood vessels, becoming the only source of oxygen supply (Laurent, 1996). The gills, in contrast, become dry and covered with a thick layer of dried mucus (Chng et al., 2016), losing almost all their function. As a result, systemic blood is preferentially allocated to the pulmonary circulation, and oxygenated blood flow is allocated mainly to the systemic circulation. Despite these impressive changes, the morphological integrity of the gills is preserved to allow rapid restoration of nitrogen metabolism and respiratory function upon arousal (Sturla et al., 2002; Chng et al., 2017b) The GO enrichment results also clearly showed that the lung is more active and becomes the main respiratory organ during the maintenance phase. In addition, as a tumor suppressor gene, *p53* mainly acts by inhibiting the cell cycle and killing problem cells (Xu and Hao, 2021) whereas cyclin regulatory genes enhance their ability to prevent cell proliferation and show upregulated expression in the gill and lung, respectively. This indicates that the two organs of aestivating lungfish have different physiological states and different regulation modes. Overall, these results suggest that lungfish can achieve organ function migration in the early stage of aestivation and indirectly validate previous results and our macroscopic findings.

### Genes involved in protein translation are upregulated in aestivating *P.annectens*

As an evolutionary choice to cope with adverse conditions, aestivation should normally be characterized by the lowest possible energy expenditure, but we also found significant upregulation of some pathways. According to our enrichment results for 56 upregulated GO terms, both gill and lung exhibited significantly high upregulation of genes related to substances required for protein biosyntheses, such as various types of RNA synthesis, ribosome synthesis, spliceosome synthesis, and RNA polymerase production, during the maintenance phase of aestivation. The same results were reported by Faherty et al. (2018) for hibernating dwarf lemurs. If our results are correct, it is clear that the upregulation of such genes during the maintenance phase is an important choice that allows for the rapid synthesis of proteins and RNAs required by the organism during the wake phase. This result is fascinating, and it makes us think about the reasons behind it and whether there is some intrinsic connection between the two states of dormancy. It is generally believed that there are two likely explanations for this result (Faherty et al., 2018). First, during aestivation protein biosynthetic machinery is recruited to actively participate in translation. Second, inhibition of the mRNA degradation pathway during aestivation results in mRNA hoarding.

Increased expression of myostatin in aestivating lungfish is effective at preventing muscle atrophy (Ong et al., 2017). mRNA and protein expression of Bhmt1 is upregulated in the liver, probably to regulate hepatic homocysteine concentrations (Ong et al., 2015). In addition, RhGP, urea transporters, and aquaporins show upregulated expression during the maintenance phase of aestivation (Chng et al., 2016; 2017a; Chng et al., 2017b). Interestingly, the upregulation of protein synthesis pathways is also seen in hibernating animals. Coordinated upregulation of protein biosynthesis genes is a distinctive feature of the transcriptome of American black bears in hibernation (Fedorov et al., 2009; Fedorov et al., 2011; Fedorov et al., 2014).

An upregulation of pathways involved in protein biosynthesis in lungfish—an energetically expensive process—would in general be surprising, as during aestivation lungfish cease feeding for up to 6 months and display whole-body metabolic suppression to conserve their limited energy stores (Blanco & Rahalinarivo, 2010; Blanco et al., 2013; Blanco et al., 2016; Faherty et al., 2016). If the first explanation above were identified as the case, it would be an important trade-off to guarantee the survival of lungfish. However, in our study, it was impossible to test this hypothesis, as we only measured levels of differential expression. In addition, statistical patterns obtained in previous experimental studies indicate a significant decrease in lungfish metabolism regardless of the conditions of entry into aestivation (Smith et al., 1930; Swan et al., 1968; Lahiri et al., 1970; DeLaney et al., 1974), and Loong *et al.* found that the decrease in metabolism was mainly due to hypoxia (Loong et al., 2008a; Loong et al., 2008b). Therefore, the more conservative hypothesis is that the second explanation—having to do with the degradation speed of mRNA transcripts—is a much more likely explanation for upregulated expression of genes during aestivation than a new transcription of protein-related genes. This is an avenue that warrants further study in our system and others *via* proteomic screens of these genes during aestivation.

### Variations in gene expression during aestivation

We also identified numerous genes that were differentially expressed in lungfish during the important stages of aestivation. For example, genes related to aerobic respiration and lipid metabolism were significantly downregulated during aestivation compared to non-aestivation, including coenzyme Q-binding protein, carbamoyl-phosphate synthetase 2, monoglyceride lipase, and so on. These results are consistent with the hypometabolism associated with entry into aestivation. However, genes such as *COX* assembly mitochondrial protein and acyl-CoA-containing binding domain 6 were significantly upregulated during aestivation (Chng et al., 2017b). The upregulation of genes related to energy metabolism suggests that aestivating lungfish must adapt to hypoxic conditions to sustain themselves.

Hypometabolism is an important response designed to reduce energy consumption in dormant animals. Simultaneously, the immune system undergoes corresponding changes. In the transcriptome of the gills and lungs of aestivating lungfish, genes related to immunity showed a change in expression compared to non-aestivating fish. As shown in Supplementary Table S4, while genes such as *CHRNB4*, *NFKB1*, and *UNC13D* were downregulated, making it difficult for neutrophils to be activated, negative regulatory genes related to lymphocyte activation, such as *ARG1*, *AXL*, and *BCL6*, were also downregulated. This positive and negative change ensured that the lungfish maintained a certain level of immunity while dormant while reducing energy consumption. Also, complement components and coagulation factors were significantly upregulated in the gills and lungs of non-aestivating *P. annectens*. These changes all reflect adjustments by the body. The complement system is essential for cellular integrity and tissue homeostasis, and the production of complement components can be divided into central (hepatic) and peripheral compartments (Zipfel & Skerka, 2009; Sacks & Zhou, 2012). The complement system was identified for its complementary bactericidal activity and its role in the phagocytosis of cellular debris (Zipfel & Skerka, 2009). Dormant animals stop ingesting food, have a reduced risk of injury, and slow their metabolism, which leads to less bacterial invasion and less production of harmful metabolites. Consequently, the downregulation of complement components and coagulation factors could conform to physiological regulation during animal dormancy.

Signal transduction plays a key role in physiological reactions. Physiological reactions caused by hypometabolism must be successfully communicated *via* signaling pathways to activate the corresponding molecules. Many genes related to signal transduction were downregulated in aestivating lungfish. For example, downregulation of the *PI3* Kinase-*Akt* signaling pathway in gills during the maintenance period of aestivation results in reduced *AKt* expression (Garofalo et al., 2015). As shown in Supplementary Table S4, the *fas* gene encodes a protein involved in signaling that leads to self-destruction (apoptosis) when cells are not needed (Cao et al., 2010). The *fas* cell surface death receptor (*fas*) is significantly downregulated in aestivating lungfish, which suggests that lungfish maintain their cell population by inhibiting apoptosis during aestivation.

In addition, expression of both ceruloplasmin and carbamoyl-phosphate synthase 1 in the lungs was reduced in both the DEG analysis and qRT-PCR experiments, in contrast to previous studies (Loong et al., 2012). The downregulation of ceruloplasmin could be the result of reduced oxygen uptake and iron transport. Ceruloplasmin, a member of the multicopper oxidase enzyme family, is a serum ferroxidase that contains more than 95% of the copper found in plasma (Hellman and Gitlin, 2002). Ceruloplasmin is synthesized in hepatocytes, but extrahepatic ceruloplasmin gene expression has been observed in many tissues, including the spleen, lung, testis, and brain (Aldred et al., 1987; Fleming & Gitlin, 1990; Klomp et al., 1996; Yang et al., 1996). Ceruloplasmin is involved in transporting iron across the cell membrane. This protein utilizes copper ions to couple substrate oxidation with four-electron reduction of dioxygen (Hellman and Gitlin, 2002). The downregulation of ceruloplasmin gene expression in the lungs of *P. annectens* aestivating in mud for 6 months could therefore be due to reductions in oxygen uptake and iron transport. Loong *et al.* indicated that *cp* gene expression was upregulated in the liver of *P. annectens* aestivating in the air for 6 days but speculated that tissue injury and inflammation could have occurred (Loong et al., 2012).

Carbamoyl-phosphate synthase 1 participates in the urea cycle, a series of reactions that occur in liver cells. It is a highly tissue-specific enzyme whose function and expression are limited to the liver and, to a lesser extent, the intestine (Ryall et al., 1986; Van Beers et al., 1998; Summar et al., 2003). The urea cycle processes excess nitrogen into urea *via* the expenditure of two ATPs, and urea is excreted by the kidney (Lusty, 1978). The excretion of excess nitrogen prevents it from accumulating in the form of toxic ammonia. In our study, *cps1* was also expressed in the lungs of freshwater *P. annectens*, and it was downregulated in the lungs of aestivating *P. annectens*. However, Long *et al* (Loong et al., 2012) observed the upregulation of *cps1* mRNA expression in the liver of *P. annectens* aestivating in the air for 6 days. There are at least two possible explanations for this discrepancy. First, *cps1* gene expression must vary by organ because of the diverse mechanisms for coping with aestivation. Second, *cps1* gene expression must vary by phase of aestivation because the nitrogen content in the body must be gradually reduced from the induction phase of aestivation to the maintenance phase.

### Potential cancer-inhibiting ability

It was previously reported that hamsters can effectively inhibit the growth of human tumors implanted in subcutaneous tissue during hibernation. This suggests that there is an unknown signal of strong dormancy in hibernating mammals. This signal can effectively inhibit the growth and development of autologous cells in these mammals. It can also be recognized and used by mammalian tumor cells that cannot hibernate on their own (Bischoff et al., 1940; Lyman and Fawcett, 1954; Patterson et al., 1957). In the last century, Swan et al. (1968) also found antimetabolic factors in the brains of aestivating African lungfish. This factor is essentially a polypeptide with certain inhibitory activity. It can effectively reduce body temperature, reduce oxygen consumption, and inhibit DNA synthesis in rats, and this process is non-toxic and reversible (Chew et al., 2015) The results we obtained, show the presence of a large number of genes for growth and development and suppression of related negative regulatory genes, as well as the activation of cell cycle control genes, can only indicate at this point that lungfish can inhibit cell growth and differentiation, reduce metabolism, and inhibit material transport in the maintenance phase of aestivation. In addition, the significant downregulation of the tumor glycoprotein pathway in both organs and the significant inhibition of various physiological processes in aestivating African lungfish indirectly suggest an increased ability among African lungfish to inhibit tumor growth during aestivation. Answering the question of whether lungfish can effectively inhibit tumor growth during aestivation will be the goal of our next work.

## Conclusion

In this study, as an important part of an extensive exploration of physiological changes during aestivation in African lungfish, we performed the first holistic and simultaneous analysis of the gills and lungs of African lungfish, sequencing the transcriptomes of non-aestivation and aestivation fish using Illumina sequencing technology. Compared to previous methods, this technology provides higher sequencing depth and more complete transcriptome coverage, which is essential to unravel the molecular mechanisms of aestivation. The different gene expression results we obtained for non-aestivation lungfish and in the gills and lungs of lungfish after 6 months of aestivation in mud cocoons. Not only do we validate the results of previous studies in terms of transcriptomic aspects, but we hypothesize from the enrichment results that aestivation and hibernation may have similar molecular mechanisms. In addition, we found many genes that deserve further exploration for differential gene expression related to aestivation mechanism. All of the above will be the focus of our next further studies on aestivation. In conclusion, these results provide new insights into African lungfish and aestivation mechanisms, and also provide some help for future exploration of the molecular mechanisms of aestivation in African lungfish.

## Data Availability

The original contributions presented in the study are publicly available. This data can be found here:PRJNA903405.
